# Development of a virulent O’nyong’nyong challenge model to determine heterologous protection mediated by a hydrogen peroxide-inactivated chikungunya virus vaccine

**DOI:** 10.1371/journal.pntd.0012938

**Published:** 2025-03-17

**Authors:** Whitney C. Weber, Zachary J. Streblow, Takeshi F. Andoh, Michael Denton, Hans-Peter Raué, Ian J. Amanna, Dawn K. Slifka, Craig N. Kreklywich, Irene Arduino, Gauthami Sulgey, Magdalene M. Streblow, Mark T. Heise, Mark K. Slifka, Daniel N. Streblow

**Affiliations:** 1 Vaccine & Gene Therapy Institute, Oregon Health & Science University, Beaverton, Oregon, United States of America; 2 Division of Pathobiology & Immunology, Oregon National Primate Research Center, Oregon Health & Science University, Beaverton, Oregon, United States of America; 3 Division of Neuroscience, Oregon National Primate Research Center, Oregon Health & Science University, Beaverton, Oregon, United States of America; 4 Najít Technologies, Inc., Beaverton, Oregon, United States of America; 5 Department of Clinical and Biological Sciences, University of Turin, Orbassano, Italy; 6 Department of Microbiology & Immunology, University of North Carolina at Chapel Hill, Chapel Hill, North Carolina, United States of America; Beijing Children's Hospital Capital Medical University, CHINA

## Abstract

O’nyong-nyong virus (ONNV) is a mosquito-transmitted alphavirus identified in Uganda in 1959. The virus has potential for enzootic and urban transmission cycles, and in humans, ONNV infection manifests as fever, rash, and joint/muscle pain that can persist. There are currently no specific vaccines or antiviral treatments for ONNV. Since highly passaged alphaviruses often lose pathogenic features, we constructed an infectious clone for ONNV-UVRI0804 (ONNV_0804_), a 2017 isolate from a febrile patient in Uganda. Viral replication for ONNV_0804_ was compared to the highly passaged strain, ONNV_UgMP30_, and ONNV_UgMP30_ replicated to higher levels in human dermal fibroblasts and Vero cells, but both viruses replicated similarly in C6/36 and mouse embryonic fibroblast cells. We performed a head-to-head comparison of *in vivo* virulence in both immunocompetent C57BL/6 mice and interferon deficient AG129 mice. In both mouse strains, ONNV_0804_ was substantially more pathogenic than ONNV_UgMP30_. Unlike ONNV_UgMP30_, ONNV_0804_ caused significant footpad swelling and broader tissue distribution with higher vRNA loads at both 5- and 43-days post-infection (dpi) relative to ONNV_UgMP30_. This finding indicates that ONNV can persist in joint and muscle tissues for long periods of time, which has been associated with chronic arthritogenic human disease. In AG129 mice, ONNV_0804_ caused a more rapid onset of disease, higher viremia, and a >800-fold increase in virulence. Previous studies have shown that CHIKV infection or vaccination can provide cross-reactive immunity to ONNV. To determine if a CHIKV vaccine can protect against the more virulent ONNV_0804_ strain, we vaccinated mice with a hydrogen peroxide-inactivated CHIKV vaccine, HydroVax-CHIKV. Neutralizing antibody titers were determined against ONNV_0804_ and CHIKV and animals were challenged with ONNV_0804_. An optimized two-dose vaccination regimen of HydroVax-CHIKV protected against lethal infection and reduced virus-associated arthritogenic disease. These data indicate that we have developed new and robust models for studying severe ONNV disease and that HydroVax-CHIKV vaccination can protect against infection with a highly pathogenic contemporary strain of ONNV.

## Author summary

O’nyong-nyong virus (ONNV) is a mosquito transmitted virus capable of causing debilitating arthritis and myalgia in humans. While the virus is currently circulating in Africa, there is a lack of FDA-approved countermeasures to prevent or treat ONNV infections and disease. Additionally, there has been a general dearth of contemporary ONNV clinical isolates to study the biology of the virus or to aid in the development of vaccines or therapeutics. We aimed to clone a 2017 ONNV strain from Uganda called UVRI0804 from a published sequence and examine the virus isolate’s ability to cause disease in mouse models. The recovered strain ONNV_0804_ replicated in several cultured cell types but had reduced *in vitro* growth when compared to ONNV_UgMP30_, a highly passaged strain also from Uganda. However, ONNV_0804_ replicated better than ONNV_UgMP30_ in both immunocompetent and immunocompromised mice. This new isolate caused significant arthritogenic disease and viral RNA was detected in joint and muscle tissues more than 40 days post infection. A hydrogen peroxide-inactivated chikungunya virus vaccine protected against infection and disease. The infectious clone for 2017 ONNV UVRI0804 clinical strain will contribute to our understanding of ONNV pathogenesis and aid in the development of anti-infectives against ONNV.

## Introduction

O’nyong-nyong virus (ONNV) is an enveloped, positive-sense, single-stranded alphavirus in the *Togaviridae* family with a high degree of similarity in genetics and clinical manifestation to chikungunya virus (CHIKV). ONNV is a neglected, emerging and re-emerging virus first isolated in 1959 [1] that has been responsible for 3 major human epidemics. The first began around 1959 with over 2 million people infected in northwest Uganda. ONNV disappeared from detection between 1962 and 1996 then a second outbreak occurred where over 21,000 people were affected between 1996 and 1997 in southern Uganda [2–4]. Another smaller outbreak occurred in 2003 in Chad and a single case was reported in 2004 [5], further demonstrating potential for periodic re-emergence [6]. Although only three major outbreaks have been recognized, numerous studies have uncovered serological evidence of ONNV transmission, including in 2020, but there is potential that some of these reports may have been detecting cross-reactive antibodies elicited by CHIKV infection rather than ONNV infection [7–10]. ONNV is transmitted by *Anopheles funestus* and *Anopheles gambiae* mosquitoes, nighttime biting mosquitos, which contrast with CHIKV transmission vectors. These mosquitoes also transmit malaria and are prevalent in many parts of Africa leading to outbreaks in West, East, and Central Africa [11]. Animal reservoirs of ONNV are currently undefined, but some serological evidence identifying antibodies in buffalo, duikers, and mandrills within the Congo basin (Gabon, Democratic Republic of the Congo) and has been reported [12]. The primary clinical symptoms of ONNV include fever, arthralgia, myalgia, and rash but fatigue, headaches, and lymphadenopathy are also common [3], which resemble those of other arboviral diseases such as CHIKV, dengue virus (DENV) and Zika virus (ZIKV) complicating clinical diagnosis and likely leading to an underestimation of the number of infected individuals. The incubation period of ONNV is typically 4-7 days followed by the acute phase lasting one to two weeks, but joint pain and fatigue have been observed to persist for several weeks to years in some individuals [13–15]. Although ONNV strain-specific differences in clinical manifestation have not yet been identified, differences in pathogenicity in mice have been noted [16]. Despite a significant impact on public health during outbreaks, ONNV remains understudied, and therapeutics to treat infections and a vaccine to prevent them are currently unavailable.

ONNV is genetically and antigenically related to CHIKV and other viruses of the Semliki Forest antigenic complex such as Ross River virus (RRV), Mayaro virus (MAYV), and Una virus (UNAV). Due to shared antigenicity, it has been demonstrated that CHIKV infection can induce ONNV-neutralizing antibodies in humans (and vice versa) [17–20] and that CHIKV infection can confer protection against ONNV challenge in mice [21]. Vaccines have been developed for CHIKV with cross-reactivity against ONNV [18,22–24] and some have been cross-protective [21,25] but no vaccines specifically targeting ONNV have been developed. In this study, we generated a full-length infectious clone from the published genome sequence designated ONNV UVRI0804 isolated in 2017 [26] to compare pathogenic features to a highly passaged strain (ONNV_UgMP30_) and to demonstrate cross-protective potential of our previously reported hydrogen peroxide-inactivated HydroVax- CHIKV vaccine [22]. In line with extensive cell culture passage, we observed modest replication advantages for ONNV_UgMP30_ *in vitro* but found ONNV_0804_ to be far more pathogenic *in vivo* in both immunocompetent and immunodeficient mice. Moreover, we found that HydroVax-CHIKV vaccination elicited ONNV_0804_ neutralizing antibodies that were cross-protective against lethal ONNV_0804_ infection and arthritogenic disease progression in mice.

## Results

### Genetics and replication comparison of ONNV strains

**Fig 1:**
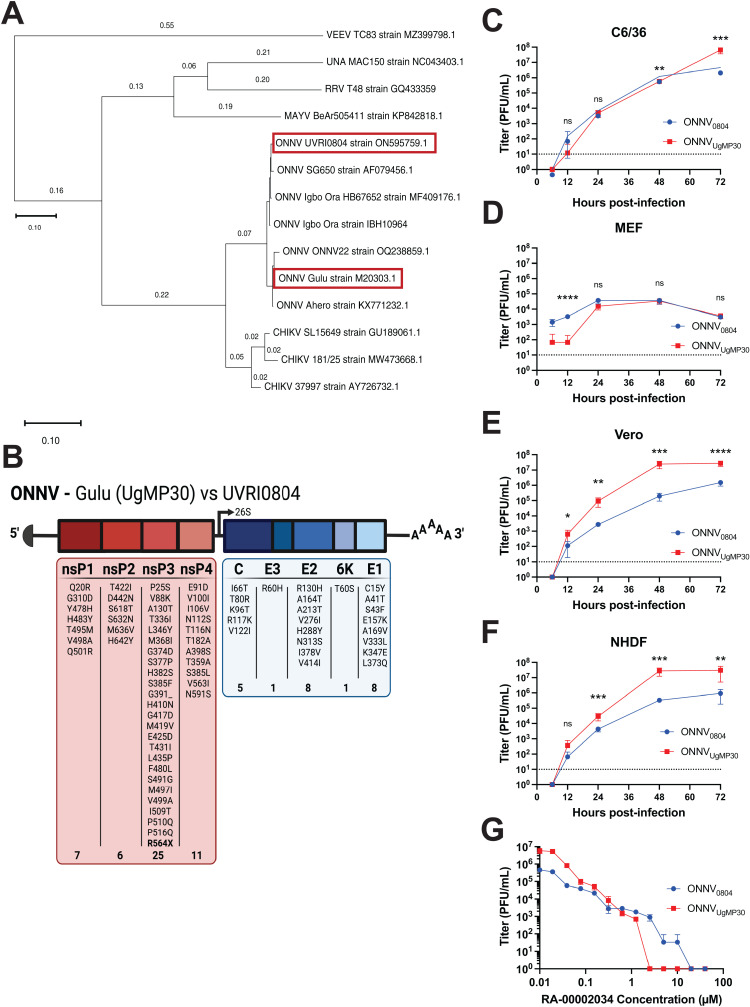
ONNV strain genetic comparison, growth characteristics in four cell lines, and antiviral inhibition. **(A)** Maximum likelihood phylogenetic tree constructed in MEGA software using the Dayhoff model and structural protein (C/E3/E2/6K/E1) amino acid sequences from all ONNV strains with available complete genomes and selected related alphaviruses of the Semliki Forest virus complex. ONNV_0804_ and ONNV_UgMP30_ strains are indicated in red outlined boxes. **(B)** Summary of amino acid differences between ONNV Gulu UgMP30 and UVRI0804 strains. The nsP3 opal stop codon is in bold lettering. Growth kinetics of ONNV_UgMP30_ (red) and ONNV_0804_ (blue) strains in **(C)** C6/36, **(D)** MEF, **(E)** Vero, and **(F)** NHDF cell lines. In three individual experiments, cells were infected at a multiplicity of infection equal to 0.5 in triplicate wells. Viral supernatants were collected at the indicated timepoints in hours post-infection (hpi) and titered by plaque assays. Titers are reported in plaque forming units (PFU) per mL of viral supernatant. The dotted line represents the limit of detection at 33.3 PFU/mL. Mean values from the three experiments and error bars with standard deviation are plotted and analyzed by multiple paired t tests with Holm-Šídák’s multiple comparisons where not significant (ns) *P* > 0.05, *P ≤ 0.05, **P ≤ 0.01, ***P ≤ 0.001, and ****P < 0.0001. **(G)** Antiviral effects of CHIKV nsp2 protease inhibitor, RA-00002034, against ONNV_0804_ and ONNV_UgMP30_ strains. NHDFs were treated with DMSO control or 2-fold serial dilutions of RA-00002034 ranging from 40-0.019 µM for 1 hour prior to infection with ONNV_0804_ or ONNV_UgMP30_ (MOI = 0.5 PFU/cell). At 2 hpi, cells were washed, and fresh medium was added containing compound. At 24 hpi, supernatants were collected, and infectious virus was quantified by plaque assay. Limit of detection was plotted as 10 PFU/mL.

While ONNV was first identified in Uganda in 1959 [1] and there have been numerous large outbreaks, relatively few viral isolates are available for research studies. In 2014, the U.S. Centers for Disease Control and Prevention and the Uganda Virus Research Institute initiated an outpatient study to identify causes of acute febrile disease in northwestern Uganda. In 2017, a sample collected from a febrile patient with fever, chills and joint pain was tested and found to cause cytopathic effect in Vero cells [26]. RNA was extracted, sequenced, and an 11kb genome aligned to the ONNV isolate, SG650, with a high degree of similarity (98.3% identical). To characterize this new isolate, named ONNV-UVRI0804, we first performed a phylogenetic analysis based on the structural proteins of the available ONNV strains with complete genomes and a selection of related Semliki Forest complex alphaviruses (Fig 1A). The ONNV strain cluster form two separate clades and are positioned between CHIKV_SL15649_ and MAYV_505411_. ONNV_UgMP30_, which is one of the more common ONNV strains used in research studies, shares 98% amino acid identity with ONNV_0804_. Alignment of amino acids revealed multiple differences between the two strains, finding more conservation in the structural proteins than non-structural proteins, with highest divergence found in nonstructural protein 3 (nsP3) (Fig 1B). Of note, ONNV_0804_ contains the opal stop codon sequence directly preceding nsP4, which has been linked to infectivity conferring a fitness advantage whereas ONNV_UgMP30_ contains an arginine residue instead [27]. To evaluate whether strain differences affect *in vitro* and *in vivo* viral replication, we used the published viral sequence for ONNV-UVRI0804 to construct a plasmid infectious clone containing the entire genome. RNA was synthesized by *in vitro* transcription, transfected into Vero cells, and the recovered recombinant virus (ONNV_0804_) was passaged in mosquito cells and sequenced by NGS to confirm genome integrity.

We compared the kinetics of viral replication for ONNV_0804_ and ONNV_UgMP30_ in four cell lines: mosquito cells (C6/36), mouse embryonic fibroblasts (MEF), African green monkey kidney epithelial cells (Vero), and primary human dermal fibroblasts (NHDF). The two viruses replicated similarly in C6/36 cells (Fig 1C) and MEFs (Fig 1D), but ONNV_UgMP30_ replicated to higher levels in Veros (Fig 1E) and NHDF cells (Fig 1F). We next wanted to examine if an antiviral compound with specificity to CHIKV could inhibit the replication of these viruses *in vitro*. The previously published CHIKV nsp2 protease inhibitor, RA-00002034, has been shown to have potent activity against a panel of alphaviruses, however it’s antiviral activity has not been evaluated against ONNV [28]. Therefore, we performed a dose response antiviral activity assay in NHDF cells against both ONNV_0804_ and ONNV_UgMP30_ with 2-fold dilutions of RA-00002034 between 40 µM-0.019 µM. The antiviral RA-00002034 demonstrated potent activity in reducing viral replication of both ONNV_0804_ and ONNV_UgMP30_ strains. Consistent with the level of potency against CHIKV, the protease inhibitor displayed a 90% inhibition concentration (IC_90_) equal to 0.021 and 0.031 µM against ONNV_0804_ and ONNV_UgMP30_, respectively (Fig 1G). These studies provide an important comparison of the growth of these strains *in vitro* and propose a potential antiviral drug candidate.

### ONNV_0804_ is more pathogenic than ONNV_UgMP30_ in immunocompetent mice

**Fig 2:**
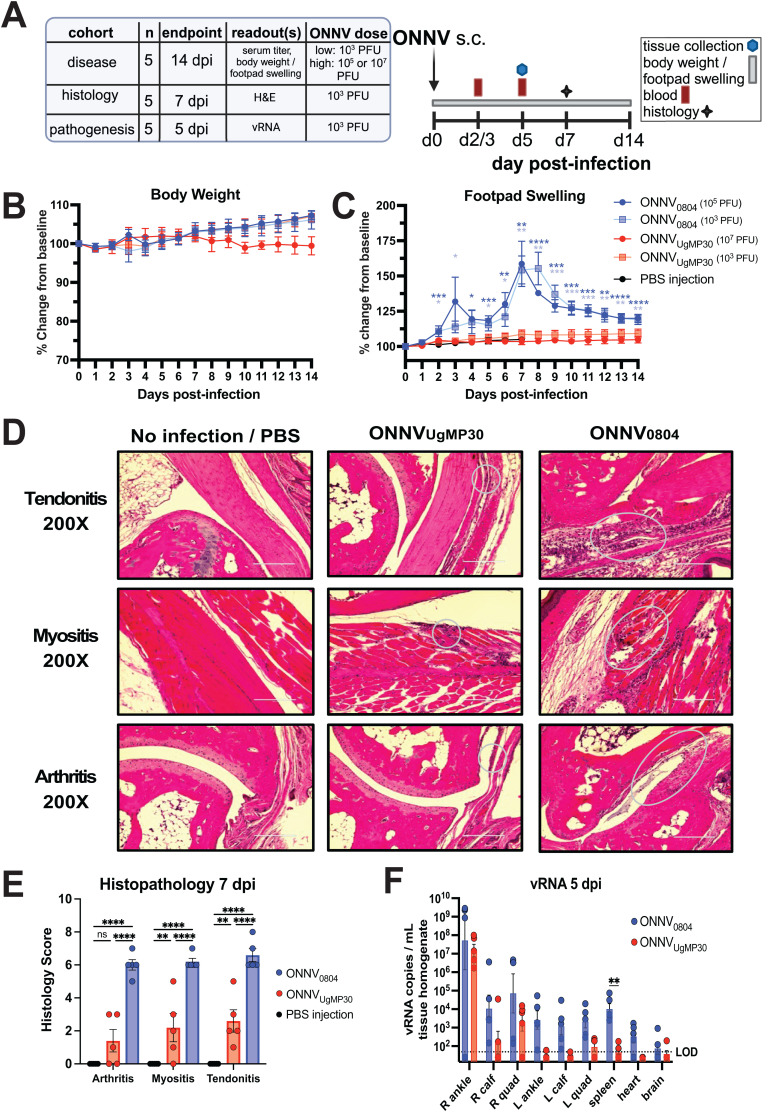
ONNV pathogenesis, disease, and viral persistence in immunocompetent mice. **(A)** Overview of study design. C57BL/6 mice (*n* = 5/group) were inoculated with a low or high dose of either ONNV strain in the right footpad (s.c.) then (**B**) body weight and (**C**) footpad swelling were monitored for 14 days. An additional group of mice (*n* = 5/group) were inoculated with 10^3^ PFU of either ONNV strain or PBS, tissues were collected at 7 dpi and perfused with 4% PFA for **(D)** H&E histological staining with ellipses designating areas undergoing inflammation and (**E**) inflammation grading on a scale of 0 to 10 with 0 indicating no inflammation and 10 indicating the most severe inflammation (see methods). Mean and SEM are plotted and analyzed by two-way ANOVA with Tukey’s multiple comparisons. **(F)** For comparison of viral replication in tissues, mice (*n* = 5/group) were inoculated with 10^3^ PFU of either ONNV strain and ankles, calf muscles, quadricep muscles, spleen, heart, and brain were collected for vRNA quantification by qRT-PCR. Data in (**F)** are log-transformed, the mean and standard error are plotted, and data are analyzed by two-way ANOVA with Šídák’s multiple comparisons where ns *P* > 0.05, *P ≤ 0.05, **P ≤ 0.01, ***P ≤ 0.001, and ****P < 0.0001. Only significant comparisons are shown.

To compare strain pathogenesis *in vivo*, we challenged immunocompetent C57BL/6 mice by subcutaneous footpad injection with two different dosages of ONNV_0804_ or ONNV_UgMP30_ (Fig 2A). Although body weight was not impacted following infection with either strain (Fig 2B), ONNV_0804,_ at a dose of 10^3^ PFU or 10^5^ PFU, caused footpad swelling in mice beginning at 2 dpi that was significantly higher than for mice challenged with 10^3^ PFU or 10^7^ PFU of ONNV_UgMP30_. The biphasic footpad swelling after ONNV_0804_ infection demonstrated peaks at 3 dpi and 7-8 dpi, depending upon initial infectious dose, and the swelling phenotype persisted until 14 dpi, the study endpoint (Fig 2C). In contrast, mice challenged with ONNV_UgMP30_, at either infectious dose, did not develop footpad swelling. Histological analysis of the ipsilateral ankle was performed at 7 dpi for a second group of C57BL/6 mice that were infected with ONNV_0804_ and ONNV_UgMP30_. Tendonitis, myositis, and arthritis with significant levels of inflammation were observed in ONNV_0804_-challenged animals with only minimal changes detected for mice infected with ONNV_UgMP30_ (Fig 2D **and** 2E). Tissue viral RNA (vRNA) was measured by quantitative RT-PCR using primers and probe specific for a region of genomic sequence common to both strains. Upon examination of viral dissemination, ONNV_0804_ demonstrated broader tissue distribution (joints, muscles, spleen, heart, and brain) and higher vRNA levels at 5 dpi relative to ONNV_UgMP30_ (Fig 2F). We also conducted a time course study to examine the kinetics of viral replication in the tissues in the first 5 days after infection (S1A Fig). ONNV vRNA was detected in serum samples for ONNV_0804_ at 1–4 dpi and peaked at 2 dpi, whereas vRNA was only detectable for ONNV_UgMP30_ at 4 dpi albeit at a lower level. (S1B Fig). Infectious virus was quantitated by plaque assays of tissue lysates from the ipsilateral ankles and both viruses followed similar growth kinetics between 1 and 4 dpi, but replication was elevated for ONNV_UgMP30_ at 5 dpi compared to ONNV_0804_ (S1C Fig). Between 1 and 5 dpi, ONNV_0804_ vRNA levels were elevated compared to ONNV_UgMP30_ at nearly all timepoints in the ankles, quadricep muscles, calf muscles, heart, and spleen (S1D-G Fig). Thus, our data confirm that the contemporary clinical isolate, ONNV_0804_, constructed using the viral sequence from a recent febrile patient, is virulent *in vivo* when delivered by subcutaneous injection and this new infectious clone causes higher pathology with increased viral load relative to ONNV_UgMP30_.

### ONNV_0804_ infection leads to persistence of viral RNA in muscle and joint tissues and the development of cross-neutralizing antibodies

**Fig 3:**
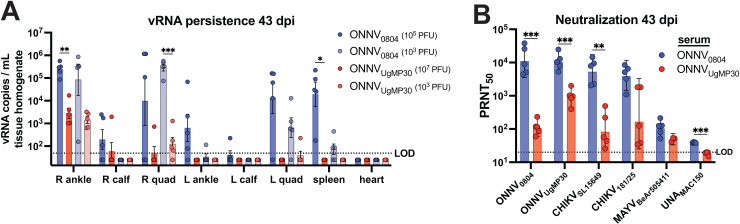
ONNV RNA persistence at 43 dpi and the development of neutralizing antibodies in immunocompetent mice. C57BL/6 mice (*n* = 5/group) were inoculated in the right footpad (s.c.) with a low (10^3^) or high (10^5^ or 10^7^ PFU) dose of ONNV_0804_ or ONNV_UgMP30_. The animals were humanely euthanized at 43 dpi for the detection of vRNA in tissues from mice challenged with each ONNV dose **(A)**. Total RNA was processed from tissue lysates and vRNA data are log-transformed; the mean and standard error are plotted. Neutralizing antibodies against ONNV and related alphaviruses by 50% plaque reduction neutralization test (PRNT_50_) in serum from mice challenged with the higher challenge doses only (*n* = 5 per group except in assays intervals against MAYV (*n* = 3) due to limited serum volume) **(B)**. Geometric mean titers (GMT) of neutralizing antibody from mice infected with 10^3^ PFU of ONNV_0804_ or 10^7^ PFU of ONNV_UgMP30_ are shown with error bars that represent 95% confidence intervals. The neutralization titers are analyzed by mixed-effects analysis two-way ANOVA with Šídák’s multiple comparisons. The vRNA persistence data are analyzed by two-way ANOVA with Šídák’s multiple comparisons. Only significant comparisons are shown in the figure (ns *P* > 0.05, *P ≤ 0.05, **P ≤ 0.01, ***P ≤ 0.001, and ****P < 0.0001).

The ability of the ONNV strains to persist long term was determined at 43 dpi in mice challenged with two doses of ONNV_0804_ (10^3^ or 10^5^ PFU) or two doses of ONNV_UgMP30_ (10^5^ or 10^7^ PFU). Ankles, calf muscles, quadricep muscles, spleen, and heart tissues were collected and processed for vRNA detection by qRT-PCR (Fig 3A). The levels of persisting vRNA were readily detected (up to ~100,000 vRNA copies/mL) in the ipsilateral ankle for both ONNV strains but ONNV_0804_ was also detected in the ipsilateral and contralateral quadricep muscles as well as the spleen. Lower levels of vRNA (~100-1,000 vRNA copies/mL) were detected in the calves and contralateral ankle for ONNV_0804_ but were below detection in ONNV_UgMP30_-challenged mice at this time point. No vRNA was detected in the heart for any animal at 43 dpi. Overall, these results demonstrate that ONNV_0804_ persists in infected tissues much more effectively than ONNV_UgMP30_, regardless of low or high dose challenge.

Serum 50% plaque reduction neutralization titer (PRNT_50_) assays were performed against viruses within the Semliki Forest virus complex using sera collected at 43 dpi from mice challenged with 10^3^ PFU of ONNV_0804_ or 10^7^ PFU of ONNV_UgMP30_ (Fig 3B). The serum PRNT_50_ values for mice infected with 10^3^ PFU of ONNV_0804_ were significantly higher against ONNV_0804_ (***P = 0.0005), ONNV_UgMP30_ (***P = 0.0009), CHIKV_SL15649_ (**P = 0.0027), and UNA_MAC150_ (***P = 0.0006) compared to serum from mice challenged with a 10,000-fold higher dose (10^7^ PFU) of ONNV_UgMP30_. Despite a high degree of sequence similarity, we detected serological differences between the two strains that are likely due to the differences in the level of viral replication competence in immunocompetent mice. Although there were differences in neutralizing antibody potency and breadth, these results demonstrated the ability of ONNV infection, with either strain, to elicit cross-neutralizing antibodies against related alphaviruses. Together these findings indicate that even in the presence of potent neutralizing antibody responses, ONNV can persist in joint and muscle tissues for long periods of time similarly to CHIKV, which has been associated with chronic arthritogenic disease [15].

### ONNV_0804_ is more pathogenic than ONNV_UgMP30_ in immunodeficient AG129 mice

**Fig 4:**
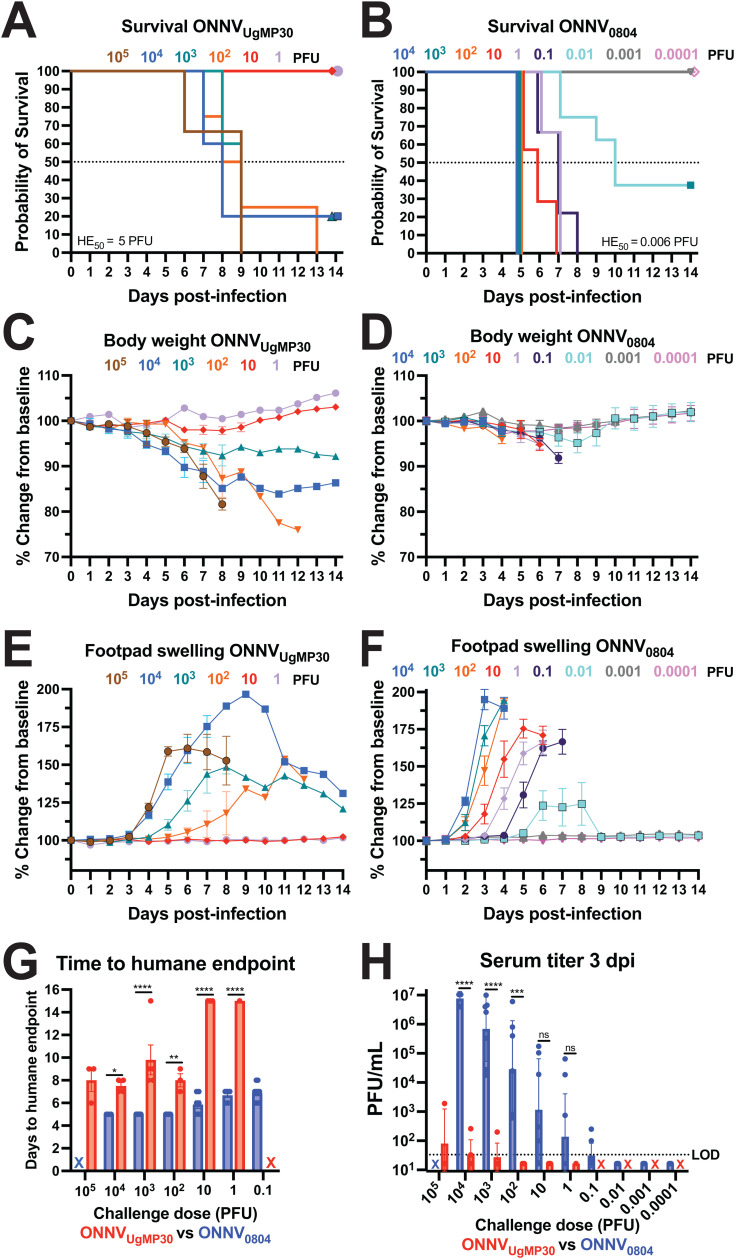
ONNV_0804_ is more virulent than ONNV_UgMp30_ in AG129 immunodeficient mice. AG129 mice were infected with a range of doses of ONNV_UgMP30_ (*n* = 3–5/dose except *n* = 1 at 1 PFU) or ONNV_0804_ (*n* = 3–9/dose) and monitored for 14 days after challenge. Kaplan-Meier survival curves for **(A)** ONNV_UgMP30_ and **(B)** ONNV_0804_ with the calculated 50% humane endpoint dose (HE_50_). The humane endpoint was defined as loss of 25% of total body weight or observance of severe lethargy. Changes in body weight over 14 days for **(C)** ONNV_UgMP30_ and **(D)** ONNV_0804_. Changes in footpad swelling over 14 days for **(E)** ONNV_UgMP30_ and **(F)** ONNV_0804_. **(G)** Comparison of time to humane endpoint compared by two-way ANOVA with Šídák’s multiple comparisons. Animals that survived infection are plotted at 15 days to humane endpoint. **(H)** Serum collected at 3 dpi was titered in triplicate by plaque assays and mean values for each mouse are plotted. The X’s in panels (**G, H**) indicate doses that were not evaluated. The LOD for this assay is 33.3 PFU/mL. Serum titers are log-transformed and analyzed by two-way ANOVA with Šídák’s multiple comparisons where ns *P* > 0.05, *P ≤ 0.05, **P ≤ 0.01, ***P ≤ 0.001, and ****P < 0.0001.

To analyze differences in pathogenicity in a more stringent disease model, AG129 mice that are deficient in alpha, beta, and gamma interferon receptors were challenged with 10^5^ PFU to 1 PFU of ONNV_UgMP30_ and 10^4^ PFU to 0.0001 PFU of ONNV_0804_. Viremia was measured at 3 dpi, and footpad swelling and body weight were quantified daily during monitoring up to the study endpoint at 14 dpi. Mice challenged with the highest dose of ONNV_UgMP30_ (10^5^ PFU) succumbed to infection between 6 and 9 dpi and the overall 50% humane endpoint (HE_50_) dose was determined to be 5 PFU (Fig 4A). Mice challenged with the highest dose of ONNV_0804_ (10^4^ PFU) succumbed to infection more rapidly and the HE_50_ was calculated to be 0.006 PFU, representing a >800-fold increase in virus-associated lethality (Fig 4B). Animals challenged with ONNV_UgMP30_ lost body weight between 5 and 12 dpi depending on the challenge dose, and some animals recovered from infection despite weight loss (Fig 4C). Mice challenged with ONNV_0804_ generally reached humane endpoint before weight loss manifested (Fig 4D). The timing of peak footpad swelling in ONNV_UgMP30_-challenged mice occurred in a dose-dependent manner, generally starting between 4 and 6 dpi (Fig 4E). Some of these animals that developed footpad swelling survived the infection. The development of footpad swelling in ONNV_0804_-challenged mice was more rapid, starting between 2 and 4 dpi, and peaked in a dose-dependent manner. Unlike ONNV_UgMP30_, each mouse that developed footpad swelling also succumbed to infection (Fig 4F). Overall, the time to humane endpoint was significantly reduced for mice challenged with ONNV_0804_ compared to ONNV_UgMP30_ (Fig 4G). ONNV_UgMP30_ viremia was not consistently detected whereas viremias for ONNV_0804_-challenged mice were significantly higher at 10^4^ PFU (*P* < 0.0001), 10^3^ PFU (*P* < 0.0007), and 10^2^ PFU (*P* < 0.0001) challenge doses. Viremia was consistently detected in all mice at 100–10,000 PFU ONNV_0804_ challenge doses. Overall, these findings affirmed that ONNV_0804_ is more virulent than ONNV_UgMP30_ *in vivo* in a susceptible mouse model of infection as evidenced by more severe disease, decreased time to humane endpoint, lower HE_50_, and higher viremia.

### HydroVax-CHIKV immunization elicits antibodies that cross-neutralize ONNV_0804_ and cross-protect against lethal arthritogenic disease in AG129 mice

**Fig 5:**
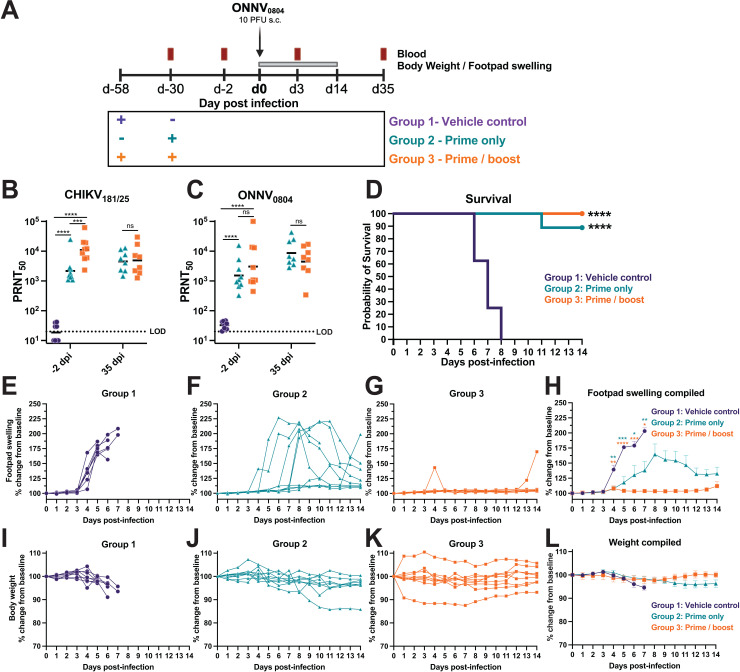
HydroVax-CHIKV immunization elicits antibodies that cross-neutralize ONNV_0804_ and cross-protect against lethal arthritogenic disease in AG129 mice. **(A)** Study schematic. AG129 mice aged 18–21 weeks old were immunized in the left leg with 0.1% Alum alone (Group 1, vehicle control, *n* = 8) or 5 μg of HydroVax-CHIKV adjuvanted in 0.1% Alum (Group 2, Prime-only, *n* = 9) at the indicated timepoints. Mice in Group 3 (Prime-boost, *n* = 9) were boosted with the same dose 28 days later. Serum was isolated two days prior to viral challenge for assessment of neutralization titers. Animals were challenged in the right footpad with 1,700 HE_50_ (10 PFU) of ONNV_0804_ then blood samples were drawn for quantification of viremia at 3 dpi by plaque assay. Animals were monitored daily for changes in footpad swelling and body weight with the humane endpoint defined as loss of 25% of total body weight or observance of severe lethargy. **(B)** Homotypic CHIKV_181/25_ neutralization and (**C**) heterotypic ONNV_0804_ neutralization titers with geometric mean shown, by 50% plaque reduction neutralization test (PRNT_50_). **(D)** Kaplan-Meier survival curve with log rank Mantel-Cox (****P < 0.0001). Changes in (**E-H**) footpad swelling and (**I-L**) body weight for each group up to 14 dpi. Compiled footpad and body weight mean with SEM are plotted in (**H**) and **(L)**, respectively. Surviving animals were humanely euthanized at 35 dpi and serum was collected to determine (**B, C**) neutralizing antibody titers. Data in **(B, C)** are log-transformed and are analyzed by two-way ANOVA with Tukey’s multiple comparisons where ns *P* > 0.05, *P ≤ 0.05, **P ≤ 0.01, ***P ≤ 0.001, and ****P < 0.0001. Data in panels (**H)** and (**L)** were analyzed by mixed-effect analysis with Dunnett’s multiple comparisons and only significant comparisons compared to group 1 controls are shown.

Utilizing the susceptible AG129 mouse model of ONNV_0804_ infection, we evaluated cross-protection elicited by a hydrogen peroxide-inactivated CHIKV vaccine (HydroVax-CHIKV) to determine whether vaccine-elicited immunity was sufficient to protect against lethal challenge with 10 PFU (1,700 HE_50_) of the highly pathogenic contemporary ONNV_0804_ strain. Mice were immunized in the left leg with 5 µg of HydroVax-CHIKV adjuvanted with 0.1% Alum (Group 2) or mock vaccinated with 0.1% Alum alone as a vehicle control (Group 1). Another group of mice received two doses of HydroVax-CHIKV with a 28-day interval in a prime-boost regimen (Group 3). As indicated in the study schematic (Fig 5A), serum was obtained at two days prior to viral challenge for the assessment of neutralizing antibody titers. Animals were challenged in the right foot pad with ONNV_0804_, blood was drawn at 3 dpi, and footpad swelling and body weight were monitored daily for up to 14 dpi. Mice that survived through the study endpoint were humanely euthanized at 35 dpi and serum was collected to assess boosting in antibody response. Sera collected two days prior and 35 days after challenge were used in neutralization assays against CHIKV_181/25_ and ONNV_0804_ to quantify homotypic and heterotypic neutralizing antibodies. The prime/boost group displayed significantly higher CHIKV_181/25_ geometric mean titers (GMT 10, 936) at −2 dpi compared with the prime-only group (GMT 2168) (*P* = 0.0014); however, the titers of these groups equalized by 35 dpi (GMT 4916 vs 4525, respectively) demonstrating boosting of antibodies in the prime-only group after ONNV challenge (Fig 5B). Similar levels of cross-neutralization activity against the heterotypic ONNV_0804_ were observed for both vaccine groups, with no significant difference found between the two vaccine groups at either time point (Fig 5C). ONNV_0804_ neutralization titers were slightly reduced compared to CHIKV_181/25_ titers prior to challenge at −2 dpi (1.4-fold lower GMT for prime-only, 3.6-fold lower GMT for prime-boost group). Unvaccinated vehicle control mice succumbed to ONNV infection rapidly and reached a 50% survival rate by 7 dpi with all mice reaching humane endpoint by 8 days after challenge (Fig 5D). The vaccinated mice in both the prime-only and prime/boost groups were protected from lethal infection with ONNV_0804_, with only one animal succumbing to infection in the prime-only group at 11 dpi (i.e., 89% and 100% protection from lethal challenge, respectively). Footpad swelling developed rapidly in the vehicle control group with onset beginning at 3 dpi and continuing to increase in thickness until reaching a humane endpoint (Fig 5E, 5H). The prime-only group had a general increase in the time to peak footpad swelling post-infection relative to the vehicle control group and many of the prime-only animals demonstrated a significant reduction between 3 and 7 dpi (Fig 5F, 5H). Indeed, a major reduction in footpad swelling was observed in many of the mice in the prime-only group, and three of the nine mice did not develop footpad swelling, indicating that the level of immunity afforded to the HydroVax-CHIKV vaccine prime-only group may be near the protective threshold for ONNV. Prime/boost vaccination prevented footpad disease in all mice except one animal which developed footpad swelling at 13–14 dpi (Fig 5G-5H). The vehicle control mice challenged with ONNV_0804_ had decreasing body weights and succumbed to infection prior to reaching the humane endpoint of 25% total weight loss (Fig 5I) while both vaccination groups were protected from major changes in weight (Fig 5J-5L). Overall, a two-dose vaccination regimen with HydroVax-CHIKV afforded effective cross-protection against heterotypic challenge with the virulent ONNV_0804_ strain in the AG129 mouse model, resulting in protection from arthritogenic disease/footpad swelling and protection from lethal infection.

**Fig 6:**
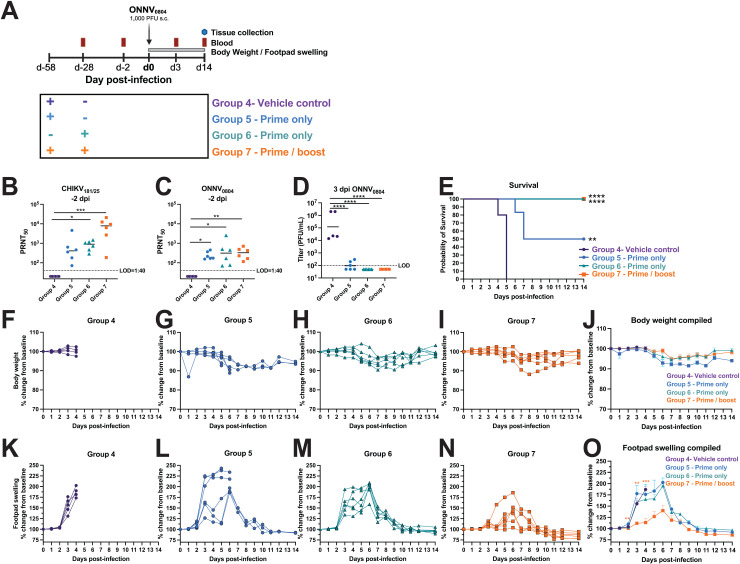
HydroVax-CHIKV vaccination partially cross-protects against 170,000 HE_50_ of ONNV_0804_ in AG129 mice. **(A)** Study schematic. AG129 mice aged 18–21 weeks old were immunized in the left leg with 0.1% Alum alone (Group 4, Vehicle control, *n* = 5) or 5 μg of HydroVax-CHIKV adjuvanted in 0.1% Alum (Groups 5 and 6, Prime-only, *n* = 6) at the indicated timepoints. Mice in Group 7 (Prime/boost, *n* = 6) were boosted with the same dose 30 days later. Serum was isolated two days prior to viral challenge for assessment of (**B**) homotypic CHIKV_181/25_ neutralization and (**C**) heterotypic ONNV_0804_ neutralization titers by PRNT_50_, geometric mean titers (GMT) are shown. Animals were challenged in the right footpad with 170,000 HE_50_ (10^3^ PFU) of ONNV_0804_ then blood samples were drawn for quantification of (**D**) viremia at 3 dpi by plaque assay. In **B-D**, GMT is shown. **(E)** Kaplan-Meier survival curve with log rank Mantel-Cox (**P = 0.0018, ****P < 0.0001). Animals were monitored daily for changes in (**F-J**) body weight and (**K-O**) footpad swelling with the humane endpoint defined as loss of 25% of total body weight or severe lethargy. For (**B-C**), data are analyzed by Kruskal-Wallis test with Dunn’s multiple comparisons and only significant comparisons are shown. In **(D)**, data are analyzed by ANOVA with Tukey’s multiple comparisons. In (**J**, **O**), data were analyzed by mixed-effect analysis with Dunnett’s multiple comparisons and only significant comparisons compared to group 4 controls are shown. Only significant comparisons are shown: ns *P* > 0.05, *P ≤ 0.05, **P ≤ 0.01, ***P ≤ 0.001, and ****P < 0.0001.

The HydroVax-CHIKV vaccine protected against lethal challenge with 10 PFU of the contemporary ONNV_0804_ strain. Next, the ability of this vaccination approach to protect against a high challenge dose of 1,000 PFU (170,000 HE_50_) was tested in AG129 mice. Animals received a single 5 μg dose of HydroVax-CHIKV vaccine either 30 days prior to challenge (Group 6) or 58 days prior to challenge (Group 5). Another group received a two-dose series of the same vaccine at 28 days apart (Group 7) and the animals were challenged at 30 days after their last vaccination (Fig 6A). Blood was drawn two days prior to challenge and was processed to test sera for neutralizing antibodies. Animals were challenged in the right footpad and blood was drawn at 3 dpi for quantification of viremia. Daily monitoring of footpad swelling and body weight measurements were conducted for all animals from 0 to 14 dpi. At 14 dpi, surviving animals were humanely euthanized for the quantification of vRNA in various tissues (Fig 6A). Sera collected two days prior to challenge were tested for homotypic and heterotypic neutralization of CHIKV_181/25_ and ONNV_0804_, respectively. The animals in prime/boost Group 7 developed a higher level of neutralizing antibodies against CHIKV_181/25_ with a geometric mean titer (GMT) of 4433 compared to the two prime only groups, Groups 5 and 6, with GMTs of 428 and 756, respectively (Fig 6B). The prime-only and prime/boost groups all developed similar levels of cross-neutralizing antibodies against ONNV_0804_ (GMT ~200-300) (Fig 6C). At 3 dpi, Group 5 had ~1,264-fold lower levels of infectious ONNV_0804_ in the serum with 3/6 animals having undetectable viremia and a geometric mean of 95 PFU/mL (****P < 0.0001) compared to Group 4 vehicle controls with geometric mean viremia of 1.2 x10^5^ PFU/mL, whereas Group 6 (****P < 0.0001) and Group 7 (****P < 0.0001) were both below the limit of detection (Fig 6D). The mock-vaccinated Group 4 vehicle control animals succumbed to infection between 4 and 5 dpi, which was more rapid than the vehicle control animals that were challenged with 10 PFU (6-8 dpi, Fig 5D). Although only 50% of Group 5 animals were protected from lethal infection (**P = 0.0018) (Fig 6E), all animals that received primary immunization at 28 days prior to challenge (Group 6) or the two-dose prime/boost regimen (Group 7) demonstrated 100% survival (****P < 0.0001) (Fig 6E). The vehicle control mice succumbed to infection by 5 dpi prior to the onset of body weight loss, whereas all vaccinated animal groups displayed minimal weight changes with slight fluctuations occurring between 5 and 11 dpi (Fig 6F-6J). Footpad swelling was significantly reduced compared to controls in only the animals in prime/boost Group 7 at 2 dpi (**P = 0.0048), 3 dpi (**P = 0.0097), and 4 dpi (***P = 0.0004) (Fig 6K, 6N **and** 6O), whereas mice in prime-only (Group 6) initially developed footpad swelling similar to the controls but was later controlled by 9 dpi (Fig 6M). Three of the animals in Group 5 showed reduced footpad swelling relative to the unvaccinated control animals in Group 4 (Fig 6L) and survived lethal ONNV_0804_ infection (Fig 6E). The other 3 animals in Group 5 appeared to have increased footpad swelling relative to the unvaccinated control animals in Group 4 at 3–4 dpi and from 5–6 dpi their footpad swelling appeared to be similar to that observed among unvaccinated vehicle control animals that survived to 6–7 dpi after infection with 100-fold lower virus dose (10 PFU; Fig 5E). At 14 dpi, all surviving animals were humanely euthanized and vRNA was quantified in the spleen, quadricep muscles, calf muscles, ankles, and heart tissue (S2 Fig). Although each of the vaccinated animals had controlled disease by 14 dpi, residual vRNA was detected in several tissues which trended higher for the Group 6 and 7 animals compared to Group 5. One caveat of this data was that a comparison could not be made to Group 4 vehicle control animals because they had all reached humane endpoint before 14 dpi. Overall, these findings indicate that the HydroVax-CHIKV prime/boost dose regimen reduced disease and confers protection in AG129 mice against a high challenge dose of ONNV_0804_.

## Discussion

ONNV is an arthritogenic alphavirus with striking similarity to CHIKV in circulation, genetics, pathogenesis, and clinical presentation. Pathogenesis of ONNV has been briefly explored *in vitro* [29] and *in vivo* in C57BL/6 [30,21] and AG129 [16,25] mouse models. Differential disease outcomes have generally not been observed in humans for different infecting ONNV strains but they have in mice [16]; although establishing a relevant infection model that has translation for human infection has been a challenge due to the minimal number of available virus stains. The UgMP30 strain has been passaged extensively in both mouse brains and Vero cells, potentially leading to reduced pathogenicity in mice. Studies have shown the SG650 [16,25] and IMTSSA/5163 [21,30] strains, both isolated from febrile patients, to be pathogenic in AG129 and C57BL/6 mice. However, these studies required high challenge doses to achieve disease in their respective models. Here, we constructed an infectious clone using the sequence of the UVRI0804 strain isolated from a febrile patient in Uganda in 2017 and compared *in vitro* replication and *in vivo* pathogenesis to the highly passaged UgMP30 strain in both C57BL/6 and AG129 mice. The two strains replicated with comparable kinetics in fibroblasts, Vero cells, and mosquito cells, but ONNV_UgMP30_ generally replicated to higher titers. We also demonstrated the efficacy of a protease inhibitor against both virus strains, proposing an effective antiviral countermeasure. In immunocompetent mice, ONNV_UgMP30_ infection failed to induce appreciable disease, whereas ONNV_0804_ caused significant footpad swelling and moderate to severe arthritis, myositis, and tendonitis. ONNV_0804_ infection led to greater viral distribution at 5 dpi and substantial levels of viral persistence at 43 dpi in several tissues including the ankle joints, quadricep muscles, and spleen. The viral persistence is consistent with what has been shown for CHIKV [31,32] and has been linked to chronic arthralgia. This finding of persistence is also consistent with a study in C57BL/6 mice, which detected luciferase tagged-ONNV-IMTSSA/5163 at 40 dpi in the infected right ankle, the site of challenge [30]. Notably, this other study required a challenge dose of 10^6^ PFU to achieve infection, disease, and viral persistence in their C57BL/6 model of ONNV infection whereas we observed these characteristics at a challenge dose of only 10^3^ PFU. Altogether, ONNV_UgMP30_ pathogenesis was reduced compared to ONNV_0804_ in C57BL/6 mice, which is consistent with previous reports of an attenuated *in vivo* phenotype [16]. This study establishes the ONNV_0804_ strain as a reproducibly virulent model of ONNV infection in both AG129 and C57BL/6 mice that can be used to evaluate vaccines and therapeutics.

A reproducible trend in pathogenicity was observed in immunodeficient AG129 mice; compared to ONNV_UgMP30_, the HE_50_ for ONNV_0804_ was >800-fold higher and the time to death, or reaching humane endpoint, was significantly reduced. In addition, the development of disease was more rapid at lower doses for ONNV_0804_. One potential explanation for the differences in pathogenesis noted between these strains could be that the ONNV_0804_ strain contains the opal stop codon between nsP3 and nsP4 whereas ONNV_UgMP30_ does not, which has been previously linked to transmissibility and infectivity fitness advantages in mosquitos [27]. Interestingly, the SG650 strain of ONNV is the only other ONNV strain containing the opal stop codon and happens to be the strain with the most comparable pathogenicity in mice to the ONNV_0804_ strain [16]. Overall, ONNV_0804_ caused similar pathogenesis in mice to what has been reported for the SG650 and IMTSSA/5163 strains but achieved this phenotype with a lower, more physiologically realistic challenge dose [16,30]. Specifically, challenge studies in C57BL/6 mice with the ONNV IMTSSA/5163 isolate collected from a febrile patient in 2004 in Chad demonstrated that a dose of 10^6^ PFU induced footpad swelling [30] and studies with the ONNV SG650 isolate caused footpad swelling disease following challenge with 10^3^ PFU in A129 mice [16]. These findings indicate that ONNV_0804_ is a more virulent and physiologically relevant isolate of ONNV in both AG129 and C57BL/6 mouse infection models, making this virus best suited for challenge studies conducted for preventative or therapeutic evaluation.

We demonstrated in both our present and previous studies [22] that the HydroVax-CHIKV vaccine elicits antibodies that cross-neutralize ONNV. We and others have shown that CHIKV infection or vaccination elicits ONNV-neutralizing antibodies [18–24], but some studies have concluded that this is a one-way antigenic relationship [33,34]. Our work in the present study comparing serological differences in alphavirus neutralization after ONNV infection with either strain (Fig 5.3B) did not reveal a one-way antigenic relationship, however, significant differences in cross-neutralization of CHIKV and other alphaviruses were identified depending on the infecting ONNV strain. For example, ONNV_0804_ infection elicited antibodies that more potently cross-neutralized strains of CHIKV compared to ONNV_UgMP30_ infection, demonstrating antigenic differences between ONNV strains. These results are surprising given the genetic similarity of ONNV strains. The differences in the antigenic profile due to infection strain may have implications for individuals susceptible to CHIKV, ONNV, and other alphaviruses, such as the degree of potential cross-protective immunity afforded by infection. Additional studies are warranted to identify differential neutralization epitopes contributing to the antigenic profile of these strains.

With CHIKV and ONNV circulation overlapping in Africa [11,35], in this study, we evaluated the cross-protective efficacy of a HydroVax-CHIKV [22] vaccine against ONNV_0804_ challenge in mice. We found that the HydroVax-CHIKV vaccine elicited ONNV-neutralizing antibodies in AG129 mice that were protective against the development of disease and increased survival following challenge with 10 or 1000 PFU. We demonstrated that a single dose of HydroVax-CHIKV provided 90% survival against ONNV challenge with 10 PFU but footpad swelling occurred in 67% of prime-only mice. Our second experiment, using a higher challenge dose, further validated this finding and revealed an impact of the prime vaccine timing on protection. In animals immunized with a single vaccine dose 58 days prior to challenge, cross-protection waned to 50% survival whereas 100% survival was observed in animals primed 28 days prior to challenge. In contrast, 100% of mice in the prime-boost group survived ONNV_0804_ challenge at both challenge doses and demonstrated significant reduction in footpad swelling, underscoring that a two-dose vaccine schedule with HydroVax-CHIKV provides effective protection against ONNV challenge. Overall, these results demonstrate the impact that cross-neutralizing antibody potency can have on cross-protection from disease, which should be carefully strategized in the design of cross-protective alphavirus vaccines.

Two studies have tested the cross-protective potential of a CHIKV-specific vaccine against ONNV. The first was reported by Partidos *et al.,* which demonstrated that one dose of an attenuated recombinant CHIKV vaccine reduced footpad swelling and weight loss and led to 100% survival of AG129 mice after 10^4^ or 10^5^ PFU ONNV_SG650_ challenge [25]. In a second study, Nguyen *et al.* showed single dose protection of a CHIKV vaccine against 10^4^ CCID_50_ of ONNV-IMTSSA/5164 viremia between 1 and 6 dpi in C57BL/6, but data exploring protection from disease was not shown because ONNV-induced disease was reportedly not observed in their model, further underscoring the relevance of using pathogenic ONNV strains for these studies [21]. Notably, in all comparable studies, a robust disease model was difficult to establish and required use of high challenge doses. Our studies build upon these findings by establishing CHIKV vaccine-mediated cross-protection against a contemporary, highly virulent strain of ONNV and provide new insights into the pathogenesis of this virus in two mouse models. Following recent U.S. Food and Drug Administration [36], Health Canada, and European Medicines Agency [37] approval of the first CHIKV vaccine [38], there are several questions regarding how CHIKV vaccine rollout will shape CHIKV and related alphaviruses transmission and distribution. Future studies should explore HydroVax-CHIKV-mediated ONNV cross-protection from viral pathogenesis and viral persistence in additional mouse models and non-human primates to better understand the mechanisms mediating protection. Our findings indicate that ONNV_0804_ is pathogenic in both immunocompetent and immunodeficient mice and that an inactivated CHIKV vaccine is capable of protecting against ONNV infection and disease in a highly susceptible mouse model.

## Materials and methods

### Ethics statement

Experiments that involved mice were performed in an Oregon Health and Science University (OHSU) ABSL-3 facility at the Vaccine and Gene Therapy Institute (VGTI). OHSU receives accreditation from the Association for Accreditation and Assessment of Laboratory Animal Care (AALAC) International. The experiments were performed in compliance with OHSU Institutional Biological Safety and the animal protocols were approved by the OHSU Institutional Animal Care and Use Committee (IACUC Protocols #0913 and 1181-02). Mice were housed in ventilated racks with access to food and water with a 12-hour light/dark cycle.

### Cells

Normal human dermal fibroblasts (NHDF; ATCC PCS-201-012) and mouse embryonic fibroblasts (MEF; ATCC BL/6-1) were cultured at 37°C and 5% CO₂ in Dulbecco’s modified Eagle medium (DMEM; Corning), supplemented with 10% fetal bovine serum (FBS; HyClone) and 1% penicillin-streptomycin-glutamine (PSG; Life Technologies) (DMEM-10). Serum-complete Vero cells (ATCC CCL-81) were cultured at 37°C and 5% CO₂ in DMEM with 5% FBS and 1% PSG (DMEM-5). *Aedes albopictus* C6/36 cells (ATCC CRL-1660) were cultured at 28°C with 5% CO₂ in DMEM-10.

### Viruses and the HydroVax-CHIKV vaccine

O’nyong’nyong virus (ONNV_UgMP30_; BEI NR-51661), Mayaro virus (MAYV_BeAr505411_; BEI NR-49910), and Una virus (UNAV_MAC150_; BEI NR-49912) were obtained from the Biodefense and Emerging Infectious Disease Research Resources Repository (BEI Resources). Chikungunya virus (CHIKV_181/25_) and CHIKV_SL15649_ were generated from infectious clones as previously described [39,40]. The O’nyong’nyong virus (ONNV_0804_) infectious clone was engineered as described below. Viral stocks were propagated in *Aedes albopictus* C6/36 cells. At 72 hours post-infection (hpi), supernatants were collected, clarified by centrifugation (Beckman CS-6, 900 x g, 15 minutes), and pelleted through a 10% sorbitol cushion by ultracentrifugation (82,755 x g for 70 minutes). Viral pellets were resuspended in phosphate buffered saline (PBS), frozen at −80°C, and titered on Vero cells using limiting dilution plaque assays in 48-well plates. Infected cells were incubated for 2 hours under continuous rocking at 37°C with 5% CO₂, then overlaid with a 2:1 mixture of DMEM-5 containing 0.3% high/low viscosity carboxymethyl cellulose (CMC-DMEM) (Sigma). Cells were fixed with 3.7% formaldehyde and stained with 0.2% methylene blue at 48 hpi for MAYV_BeAr505411_, UNAV_MAC150_, and CHIKV_SL15649_, or at 72 hpi for ONNV_UgMP30_, ONNV_0804_, and CHIKV_181/25_. Plaques were visualized under a dissecting microscope, and counts were used to calculate viral titers in plaque-forming units (PFU) per mL. Virus stocks for all experiments were passaged 1 or 2 times and were sequence-validated as described below.

The HydroVax-CHIKV vaccine was produced as previously described [22]. Briefly, CHIKV_181/25_ was propagated on serum-free Vero cells, and harvests were clarified and treated with Benzonase to minimize host-cell DNA/RNA contamination prior to concentration and buffer exchange using tangential flow filtration (TFF) followed by CaptoCore 700 chromatography (Cytiva). HydroVax-based inactivation conditions were optimized for CHIKV_181/25_ and included 0.0003% H_2_O_2_, 2 μM CuCl_2_, 20 μM methisazone, and 0.06% formaldehyde in a buffer matrix with 150 mM Na_2_HPO_4_ at pH 7.5, for 48 hours at room temperature. After inactivation, chemical components were removed using TFF. Complete inactivation was confirmed through cell culture-based residual live virus testing. HydroVax-CHIKV (5 µg/dose) was adjuvanted with 0.1% aluminum hydroxide (Alhydrogel, InvivoGen).

### Cloning strategy

To assemble the infectious clone of the O’nyong’nyong virus strain ONNV_0804_, seven genome fragments, each approximately 1700 base pairs (bp) with 20 bp of overlapping sequence, were synthesized by Twist Bioscience based on the sequence (accession number ON595759). The plasmid pSinRep5 (Invitrogen) was used as a template to generate a 2200 bp fragment using standard PCR conditions. We combined 200 femtomoles of each fragment with an equal volume of NEBuilder HiFi master mix (NEB) according to the manufacturer’s instructions. Assembly was performed at 50°C for 60 minutes. TOP10 competent cells (Invitrogen) were then transformed with 5µL of the assembled product. After DNA purification, the infectious clone (ONNV_0804_ ic) was verified by whole plasmid sequencing (Eurofins). The ONNV_0804_ ic was linearized with *Not*I digestion and transcribed *in vitro* using the SP6 mMessage mMachine kit (Invitrogen) followed by purification with the RNeasy Mini Kit (Qiagen). Vero cells were transfected with 10µg of RNA and 6 µL of Lipofectamine 2000 per well of a 6-well plate, following the Invitrogen protocol. After 3 days, supernatant was collected and stored at −80°C. Virus stocks were prepared using 100 µL of the resulting p0 stock for each T-175 flask of C6/36 cells. Viral RNAs were confirmed by Next Generation Sequencing (NGS).

### Growth curves

C6/36, MEF, Vero, and NHDF cells were seeded into 48-well plates at 2 x 10⁵ cells/well and incubated overnight at 37°C with 5% CO₂. Cells were infected in triplicate wells with either ONNV_UgMP30_ or ONNV_0804_ at an MOI of 0.1. Infection occurred in 100 µL of DMEM-5 with continuous rocking for 2 hours at 37°C with 5% CO₂. The infection media was then removed, and cells were washed twice with 500 µL of PBS and resuspended in 250 µL of DMEM-5. The supernatant was sampled for timepoints taken at 6, 12, 24, and 48 hours for PFU/mL quantification by plaque assays on Vero cells. The growth curves for C6/36 cells, Vero cells and NHDFs were averaged across three independent experiments and the average data was graphed and analyzed using Prism software.

### Protease inhibitor antiviral assay

NHDF cells were seeded into 48-well plates at 2 x10^5^ cells/well and incubated overnight at 37°C with 5% CO₂. Using a 10mM stock solution, a 12-point dilution series of RA-00002034 was prepared by diluting the compound 1:1 in DMEM-5. Media only and DMSO controls were also included. Prior to infection, 200 µL of the dilution series was added to triplicate wells of the NHDFs and incubated for 1 hour. Cells were then infected with an MOI of 0.5 with either ONNV_UgMP30_ or ONNV_0804_ in 100 µL of DMEM-5 with continuous rocking for 2 hours at 37°C with 5% CO₂. Infection media was then removed, and cells were washed twice with 200 µL of PBS and subsequently treated again with 200 µL of the dilution series. The supernatant was sampled at 24 hours for PFU/mL quantification by plaque assay on Vero cells. IC_90_ values were calculated using GraphPad Prism 10.2.3 software after log transformation and normalization of the data. A nonlinear fit analysis was then performed with F constrain equal to 90.

### Mouse experiments

C57BL/6 purchased from Jackson Laboratories and AG129 mice bred at OHSU were housed in ventilated racks with free access to food and water in a room with a 12-hour light/dark cycle. For viral challenge studies, mice were inoculated in the right posterior footpad with a 20µL subcutaneous (s.c.) injection of ONNV_UgMP30_ or ONNV_0804_ diluted in PBS. For AG129 mice, 50% humane endpoint dose (HE_50_) was calculated used the methods of Reed and Muench [41]. Vaccination experiments were performed with challenge doses of 10 or 1000 PFU and all other challenge experiments were performed with various challenge doses as indicated. Animals at 8–12 weeks of age were vaccinated by intramuscular (i.m.) injection with 5µg of HydroVax-CHIKV or 0.1% Al adjuvant in TFF buffer alone in the left leg. Serum was isolated from the saphenous vein at the indicated timepoints for measurement of neutralizing antibodies or viremia. Serum was collected from clotted blood samples after centrifugation for 5 minutes at 9000 x g. Animals were challenged at 18–21 weeks of age and footpad swelling was measured with digital calipers and changes in body weight were recorded daily for up to 14 dpi. Humane endpoint was defined as 25% body weight loss, but animals were also euthanized if they appeared severely lethargic. As indicated, ankles, quadricep muscles, calf muscles, heart, spleen, and brain were collected to assess viral dissemination.

### Histopathological analysis

At 7 dpi, PBS-control, ONNV_UgMP30_, and ONNV_UVRI0804_ infected mice were sacrificed and perfused with 4% paraformaldehyde in PBS. Lower hind legs were collected, fixed in 4% paraformaldehyde, decalcified, embedded in paraffin, and sectioned into 5-micron thick slices. Sections of ipsilateral and contralateral legs were stained with H&E and evaluated for inflammation and tissue disease by light microscopy (Olympus VS120 Virtual Slide Microscope). Pathology specialists blindly scored the histological lesions, including necrosis, inflammation, fibrosis, edema, and vasculitis, using a 0–10 scoring system: 0 (no lesions), 1–2 (minimal, 1–10% affected), 3–4 (mild, 11–25% affected), 5–6 (moderate, 26–50% affected), 7–8 (marked, 51–75% affected), 9–10 (severe, >75% affected).

### Viral RNA detection

RNA was isolated from 300 µL of each mouse tissue homogenate collected in 1 mL of PBS. Total nucleic acids were extracted using the Promega Maxwell 48 sample RSC automated purification system and the Maxwell RSC Viral TNA extraction kit. RNA was resuspended in 70 µL of RNase-free water and diluted to 100 ng/µL. Contaminating DNA was removed using ezDNase. Single-stranded cDNA was synthesized from 1 µg of total RNA using random hexamers and reverse transcriptase (Invitrogen Superscript IV) following the manufacturer’s protocol. Quantitative RT-PCR was performed on a QuantStudio 7 Flex system using the following primers and probe for ONNV RNA: Forward-CCCACAGCATGGCAAAGAAC, Reverse-CTGGCGGCATATGCACTTCT, and probe FAM-ACGTACGTCCATACCACAG–MGB. All reactions were performed in triplicate, and data were analyzed using Applied Biosystems software. Viral RNA levels were normalized to the murine housekeeping gene ribosomal protein RPS17 and reported per 1 mL of tissue homogenate.

### Quantification and isolation of infectious virus

Limiting dilution plaque assays were used to quantify infectious virus in mouse serum, tissue homogenate, or growth curve cell supernatants. Briefly, 20 µL of sample was added to 180 µL DMEM-5 for 1:10 serial dilutions. Viral dilutions were added to confluent monolayers of Vero cells in 48-well plates and incubated for 2 hours at 37°C with 5% CO₂ with continuous rocking, followed by addition of CMC-DMEM-5 overlay. Plaque assays were fixed and stained as described above.

### Neutralization assays

Mouse serum was heat-inactivated for 30 minutes at 56°C and serially diluted 2-fold in DMEM-5. Diluted serum was mixed with media containing approximately 70–120 PFU of ONNV_0804_, ONNV_UgMP30_, CHIKV_SL15649_, CHIKV_181/25_, MAYV_BeAr505411_, or UNAV_MAC150_. Mixtures were incubated for 2 hours at 37°C with 5% CO₂ with continuous rocking, then transferred to 12-well plates of confluent Vero cells. Cells were incubated for an additional 2 hours at 37°C with 5% CO₂ with continuous rocking, followed by addition of CMC-DMEM-5 overlay. Plates were incubated for 48 hours for CHIKV_SL15649_, MAYV_BeAr505411_ and UNAV_MAC150_, or 72 hours for ONNV_UVRI0804_, ONNV_UgMP30_, and CHIKV_181/25_. Cells were fixed and stained as described for plaque assays. Plaques were enumerated under a dissecting microscope or by eye depending on size and percent neutralization was determined at each dilution relative to control wells without serum.

### Statistical analysis

Data were analyzed using GraphPad Prism 10.2.3 software. Mixed-effects analyses were used to address instances of missing values. The 50% plaque reduction neutralization titers (PRNT_50_) were calculated by variable slope, non-linear regression analysis. Kaplan-Meier survival curves were analyzed by log rank Mantel-Cox. Footpad swelling and body weight changes, vRNA levels, and neutralization titers at multiple timepoints were analyzed by two-way ANOVA with Šídák’s or Tukey’s multiple comparisons. The neutralization and serum infectious titer data presented for single timepoints in Fig 6 are analyzed by Kruskal-Wallis test with Dunn’s multiple comparisons.

## Supporting information

S1 Fig ONNV viral load in C57BL/6 mice between 1 and 5 days after infection.
**(A)** Study schematic. C57BL/6 mice were inoculated with 10^3^ PFU of either ONNV strain and the indicated numbers of mice were euthanized for tissue harvest between 1 and 5 days after challenge. (**B**) Serum was collected and vRNA was quantified by qRT-PCR for 1–4 dpi. (**C**) Ankles, quadriceps, calves, heart, and spleen were collected and processed for titering by plaque assays and (**D-G**) quantifying vRNA by qRT-PCR for 1–5 dpi. Only right ankle titers are shown; no other tissues had detectable infectious virus.
(EPS)

S2 Fig Viral RNA levels relating to main Fig 6.
At 14 dpi, surviving animals were humanely euthanized for quantification of vRNA in the spleen, ankles, quadricep muscles, calf muscles, and heart. Data are analyzed by two-way ANOVA with Tukey’s multiple comparisons. Only significant comparisons are shown, **P = 0.0088.
(EPS)
